# A model for handling cell stress

**DOI:** 10.7554/eLife.22850

**Published:** 2016-11-29

**Authors:** Laura Le Breton, Matthias P Mayer

**Affiliations:** Center for Molecular Biology Heidelberg University, DKFZ-ZMBH-Alliance, Heidelberg, Germany; Center for Molecular Biology Heidelberg University, DKFZ-ZMBH-Alliance, Heidelberg, GermanyM.Mayer@zmbh.uni-heidelberg.de

**Keywords:** heat shock response, Hsf1, Hsp70, phosphorylation, chaperone, systems modeling, *S. cerevisiae*

## Abstract

The heat shock response in yeast is regulated by the interaction between a chaperone protein and a heat shock transcription factor, and fine-tuned by phosphorylation.

**Related research article** Zheng X, Krakowiak J, Patel N, Beyzavi A, Ezike J, Khalil AS, Pincus D. 2016. Dynamic control of Hsf1 during heat shock by a chaperone switch and phosphorylation. *eLife* **5**:e18638. doi: 10.7554/eLife.18638

Cells are subjected to frequent assaults either from their environment or as a consequence of development or disease. Such stresses – which can be caused by a range of conditions, including changes in temperature and mechanical stresses – are damaging to proteins, so cells mount the so-called heat shock response. This response is an evolutionarily conserved transcriptional program that is orchestrated in eukaryotic cells by a protein called Hsf1 (short for heat shock factor 1; Morimoto, 1998). Three copies of this protein combine to form trimers that bind to the promoters of Hsf1-dependent genes, which leads to higher levels of heat shock proteins inside the cell. These proteins include the chaperones that refold misfolded proteins or target them for degradation. Once the effects of the stress have been dealt with, cells reduce the production of heat shock proteins to normal levels.

The heat shock response has been studied for more than 30 years, mainly in yeast and animal cells, and different models have been proposed to explain it (Anckar and Sistonen, 2011). The intrinsic response model, which assumes that Hsf1 directly senses increasing temperature (and potentially other stresses), relies on Hsf1 transitioning from a monomer to a trimer (Zhong et al., 1998; Hentze et al., 2016). This model can only apply to animal cells because Hsf1 forms trimers in yeast under all conditions, even in the absence of heat shock (Sorger et al., 1987).

The chaperone titration model assumes that Hsf1 is kept inactive in unstressed cells by its interactions with chaperones; the presence of misfolded proteins then activates Hsf1 by attracting (or “titrating”) the chaperones away from Hsf1. Although there is evidence to support such a model, it was unclear which – if any – of the chaperones is the main regulator of the Hsf1 activity cycle. (Shi et al., 1998; Rabindran et al., 1994; Guo et al., 2001). In addition, Hsf1 is subject to a large number of post-translational modifications, such as phosphorylation, but the influence of these modifications on the heat shock response is a topic of controversy (Budzyński et al., 2015; Xia and Voellmy, 1997).

Now, in eLife, David Pincus and coworkers from the Whitehead Institute for Biomedical Research, Boston University and Harvard University – including Xu Zheng and Joanna Krakowiak as joint first authors – report how they have used a combination of mathematical modeling and cell biology experiments in yeast to address these issues (Zheng et al., 2016).

Initial experimental results demonstrated that a chaperone called Hsp70 – which is thought to damp down the heat shock response – binds to Hsf1 under non-stress conditions and is released upon a sudden increase in temperature. From these findings, Zheng et al. simulated how the expression of Hsf1-dependent genes changes in response to interactions between Hsp70, Hsf1 and misfolded proteins. Further support for the chaperone titration model came from experiments in which yeast cells expressed two types of Hsf1: wild-type Hsf1 and “decoy” Hsf1 (which can bind to Hsp70 but cannot activate the transcription of the Hsf1-dependent genes). These data are the strongest evidence to date that Hsp70 feedback regulates the heat shock response by directly associating with Hsf1, as happens in the chaperone titration model (Figure 1).

**Figure 1.**
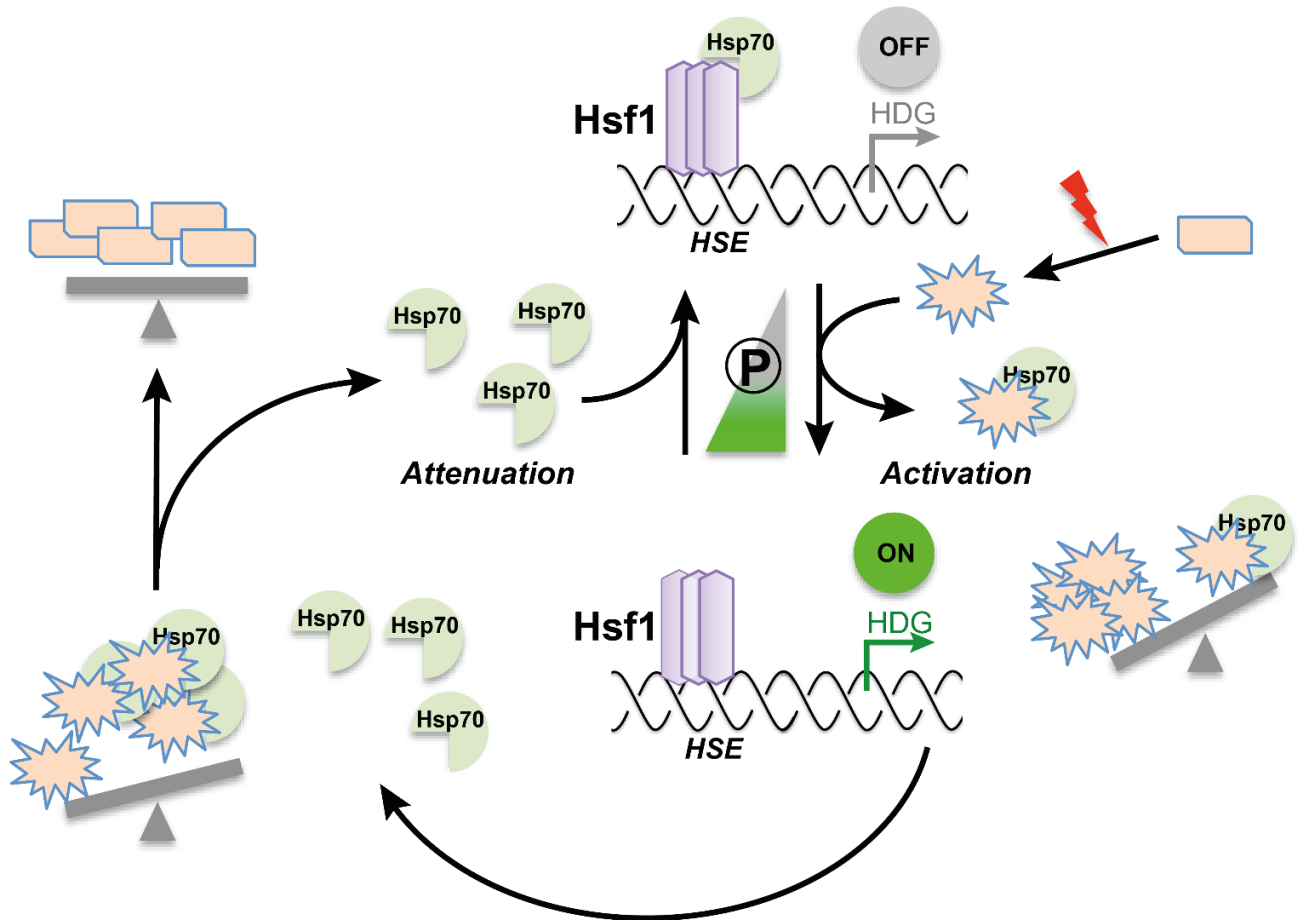
The chaperone titration model of the heat shock response. Clockwise from top: The chaperone protein Hsp70 binds to the heat shock transcription factor Hsf1, repressing its transcriptional activity. Upon a sudden increase in temperature or other stresses (red lightning bolt), fewer proteins maintain their correct shape (rectangles); misfolded proteins (stars) therefore accumulate in the cell. These misfolded proteins draw Hsp70 away from Hsf1, activating its transcriptional activity. As a result, more Hsf1-dependent genes (HDG) are expressed, leading to an increase in the number of chaperones and proteases – among them Hsp70 – in the cell. The action of the chaperones and proteases ensures that proteins can be correctly folded again; this also liberates Hsp70, which can then repress Hsf1. Middle: Hyperphosphorylation of Hsf1 (the width of the triangle represents the extent of phosphorylation) partially activates Hsf1 and sensitizes the regulatory feedback circuit.

The overexpression of Hsf1 under otherwise unstressed conditions impairs the growth of yeast cells by over-activating the transcription of Hsf1-dependent genes. Elevating the levels of Hsp70 or one of its co-chaperones in such cells rescues growth and represses the transcription of Hsf1-dependent genes. In contrast, Hsp90 – a chaperone that was believed to repress Hsf1 under non-stress conditions (Duina et al., 1998; Zou et al., 1998) – had no effect on growth or the transcription of Hsf1-dependent genes in these cells. This argues against Hsp90 playing a major role in down-regulating Hsf1 activity. Thus, the effects of the down-regulation of Hsp90 or its co-chaperones on the heat shock response might be indirect in yeast.

Zheng et al. also investigated how the hyperphosphorylation of Hsf1 helps to regulate the heat shock response. They performed *en masse* mutations of Hsf1 by either removing all 152 potential phosphorylation sites, or mimicking phosphorylation at up to 116 sites. Unexpectedly, completely abolishing phosphorylation only mildly reduces Hsf1 activity upon heat shock, indicating that phosphorylation *per se* is not required to activate Hsf1. On the other hand, mimicking hyperphosphorylation activates Hsf1 even under non-stress conditions. Moreover, the activity of Hsf1 increases further upon heat shock, which means that hyperphosphorylation only partially overwrites the repression of Hsf1 by Hsp70. Phosphorylation is thus a dose-dependent mechanism for fine-tuning the activity of Hsf1 that mainly occurs after the initial phase of the heat shock response, enhancing and prolonging it. This also means that if the stress response is activated for developmental or other reasons, the cell is still able to react to acute assaults.

It will be important in the future to assess whether these findings also apply to animal Hsf1, which interacts with chaperones such as Hsp70 and Hsp90 while in its inactive monomeric form under non-stress conditions (Shi et al., 1998; Zou et al., 1998). It also remains to be discovered how these chaperones integrate with the ability of Hsf1 to directly sense stress (Zhong et al., 1998; Hentze et al., 2016).
